# Supplementary material for: Up-regulation of cryptochrome 1 gene expression in cotton bollworm (Helicoverpa armigera) during migration over the Bohai Sea
Source: PeerJ. 2019 Nov 15;7:e8071. doi: 10.7717/peerj.8071 (PMC6859876; doi:10.7717/peerj.8071)
Supplement: Supplemental Information 1 [file peerj-07-8071-s001.docx]

Table S1. Listing of primers used in this study

| Primer name | Primer Sequences(5’-3’) | Gene |
| --- | --- | --- |
| CRY1F1 | GAYGGAGARACWGCHGGTAC | Degenerate primers for CRY1 |
| CRY1R1 | MGCGCACTCGCCMGAGTCCA |  |
| CRY1F2 | TTCGAGCARGAYTGYGAGCC |  |
| CRY1R2 | CCVGCRCASACCGACCAATC |  |
| CRY2F1 | AAGCACAYYGTVCACTGGTT | Degenerate primers for CRY2 |
| CRY2R1 | ACCCAYGGCTCRTGDATGTA |  |
| CRY2F2 | GTYGGCATYAATAAATGGAG |  |
| CRY2R2 | AGCCACATCCACATSCCAGC |  |
| CRY13RF1 | TCAAGTTTGGTAGCCTGCCCGAGTGC | 3’RACE of CRY1 |
| CRY13RF2 | CTGCACTGCGGTTTGGATGTCTTTCA |  |
| CRY15RR1 | AGCACTCGGGCAGGCTACCAAACTT | 5’RACE of CRY1 |
| CRY15RR2 | GACAGTCTCGGGCTCCCACAGAGTATG |  |
| CRY23RF1 | TGGGGGGAGTTTTTCTATTGTGCCG | 3’RACE of CRY2 |
| CRY23RF2 | GCTTGCTTCCTGACGAGAGGCGACT |  |
| CRY25RR1 | CAATGGCGTGGTGGCTCCGTTTAG | 5’RACE of CRY2 |
| CRY25RR2 | GCGATTGCGGTGTCATTTTGGGTCTC |  |
| FullC1F1 | ATTCGTCTCTCAGACCAGAGGC | Full-length primers for CRY1 |
| FullC1F2 | CCGATGTAAATATTTTGTACAGC |  |
| FullC1F3 | CGCGATAATGTTTACGGAAGGC |  |
| FullC1R1 | GCTCTTATTCTTTGAAATGGCTG |  |
| FullC1R2 | GGAATAGGAACTTCAATACCACG |  |
| FullC1R3 | GAGACACCCTGATACACAAATAC |  |
| FullC2F1 | GCATTGTTGATCTGAACTTCG | Full-length primers for CRY2 |
| FullC2F2 | GCTTCTGTGATTACCTCCTGC |  |
| FullC2R1 | TAGGTCAACGATGAGAAAAGC |  |
| QCRY1F | GATGACCTGGACAGCCAGTTC | Primers of Real-time PCR for CRY1 |
| QCRY1R | CCGAATTCCTCCCAAAGTCTT |  |
| Probe-CRY1 | AAGTTTGGCGGCAGG |  |
| QCRY2F | GCACACTAAATCCCAATG | Primers of Real-time PCR for CRY2 |
| QCRY2R | TGCTGGTTATAATTGAGTTC |  |
| Probe-CRY2 | AATGTCATGCAGTCATCACCAAGC |  |
| QactinF | CTGGGACGATATGGAGAA | Primers of Real-time PCR for actin |
| QactinR | CGAACATGATCTGTGTCA |  |
| Probe-actin | CACCACACCTTCTACAACGAGC |  |
| ActinF | CATCTACGAGGGTTACGC | Detection of templates |
| ActinR | CATCTGTTGGAAGGTGGA |  |
