# Supplementary material for: Up-regulation of cryptochrome 1 gene expression in cotton bollworm (Helicoverpa armigera) during migration over the Bohai Sea
Source: PeerJ. 2019 Nov 15;7:e8071. doi: 10.7717/peerj.8071 (PMC6859876; doi:10.7717/peerj.8071)
Supplement: Supplemental Information 2 [file peerj-07-8071-s002.docx]

Table S2. Listing of insect species used to determine selection pressures based on *cry1s* and *cry2s* sequences

| Insect specie | Genes | GenBank accession no. |
| --- | --- | --- |
| *Agrotis ypsilon* | *cry1* | JQ616846 |
|  | *cry2* | JQ616864 |
| *Mythimna separate* | *cry1* | JX077108 |
|  | *cry2* | JX077109 |
| *Bombyx mori* | *cry1* | NM_001195699 |
| *Danaus plexippus* | *cry1* | AY860425 |
| *Spodoptera exigua* | *cry1* | HQ234484 |
| *Helicoverpa armigera* | *Hacry1* | GQ896502 |
| *Mamestra brassicae* | *cry1* | AY947639 |
| *Drosophila melanogaster* | *cry1* | (NM_169852 |
| *Acyrthosiphon pisum* | *cry1* | NM_001171061 |
| *Bombyx mori* | *cry2* | NM_001195698 |
| *Helicoverpa armigera* | *Hacry2* | GQ896503 |
| *Danaus plexippus* | *cry2* | DQ184682 |
| *Apis mellifera* | *cry2* | NM_001083630 |
