# Supplementary material for: Up-regulation of cryptochrome 1 gene expression in cotton bollworm (Helicoverpa armigera) during migration over the Bohai Sea
Source: PeerJ. 2019 Nov 15;7:e8071. doi: 10.7717/peerj.8071 (PMC6859876; doi:10.7717/peerj.8071)
Supplement: Supplemental Information 3 — The length of aligned fragments and the numbers of variant nucleotides were shown in bracket. [file peerj-07-8071-s003.docx]

Table S3. Nucleotide identities among ORFs of the *Hacry1* from *H. armigera*. The length of aligned fragments and the numbers of variant nucleotides were shown in bracket.

| Gene names | Hacry1 | Hacry1-2 | Hacry1-3 | Hacry1-4 | Hacry1-5 | Hacry1-6 |
| --- | --- | --- | --- | --- | --- | --- |
| Hacry1-1 |  |  |  |  |  |  |
| Hacry1-2 | 0.955 (69/1587) |  |  |  |  |  |
| Hacry1-3 | 0.953 (72/1587) | 0.950 (79/1587) |  |  |  |  |
| Hacry1-4 | 0.925 (61/1587) | 0.922 (68/1587) | 0.922 (67/1587) |  |  |  |
| Hacry1-5 | 0.961 (60/1587) | 0.957 (67/1587) | 0.957 (68/1587) | 0.961 (3/1587) |  |  |
| Hacry1-6 | 0.998 (2/1587) | 0.957 (68/1587) | 0.954 (72/1587) | 0.927 (60/1587) | 0.962 (59/1587) |  |
| Hacry1 | 0.921 (69/1587) | 0.91 (87/1587) | 0.916 (78/1587) | 0.958 (68/1587) | 0.922 (67/1587) | 0.922 (67/1587) |
