# Supplementary material for: Up-regulation of cryptochrome 1 gene expression in cotton bollworm (Helicoverpa armigera) during migration over the Bohai Sea
Source: PeerJ. 2019 Nov 15;7:e8071. doi: 10.7717/peerj.8071 (PMC6859876; doi:10.7717/peerj.8071)
Supplement: Supplemental Information 4 — The length of aligned fragments and the numbers of variant nucleotides were shown in bracket. [file peerj-07-8071-s004.docx]

Table S4. Nucleotide identities among ORFs of the *Hacry2* from *H. armigera*. The length of aligned fragments and the numbers of variant nucleotides were shown in bracket.

| Gene names | Hacry2-1 | Hacry2-2 | Hacry2-3 | Hacry2-4 | Hacry2-5 | Hacry2-6 | Hacry2-7 |
| --- | --- | --- | --- | --- | --- | --- | --- |
| Hacry2-1 |  |  |  |  |  |  |  |
| Hacry2-2 | 0.997 (5/1935) |  |  |  |  |  |  |
| Hacry2-3 | 0.563 (1/1092) | 0.562 (4/1092) |  |  |  |  |  |
| Hacry2-4 | 0.494 (6/963) | 0.493 (9/963) | 0.875 (7/963) |  |  |  |  |
| Hacry2-5 | 0.809 (13/1935) | 0.808 (16/1935) | 0.456 (9/1092) | 0.400 (12/963) |  |  |  |
| Hacry2-6 | 0.807 (18/1935) | 0.806 (21/1935) | 0.455 (12/1092) | 0.399 (14/963) | 0.994 (13/2373) |  |  |
| Hacry2-7 | 0.786 (14/1935) | 0.785 (17/1935) | 0.443 (9/1092) | 0.388 (14/963) | 0.968 (9/2373) | 0.965 (16/2373) |  |
| Hacry2 | 0.639 (19/1536) | 0.638 (21/1536) | 0.287 (12/693) | 0.232 (14/564) | 0.821 (25/1974) | 0.818 (32/1974) | 0.798 (26/1974) |
