# Supplementary figures and images for: Up-regulation of cryptochrome 1 gene expression in cotton bollworm (Helicoverpa armigera) during migration over the Bohai Sea
Source: PeerJ. 2019 Nov 15;7:e8071. doi: 10.7717/peerj.8071 (PMC6859876; doi:10.7717/peerj.8071)

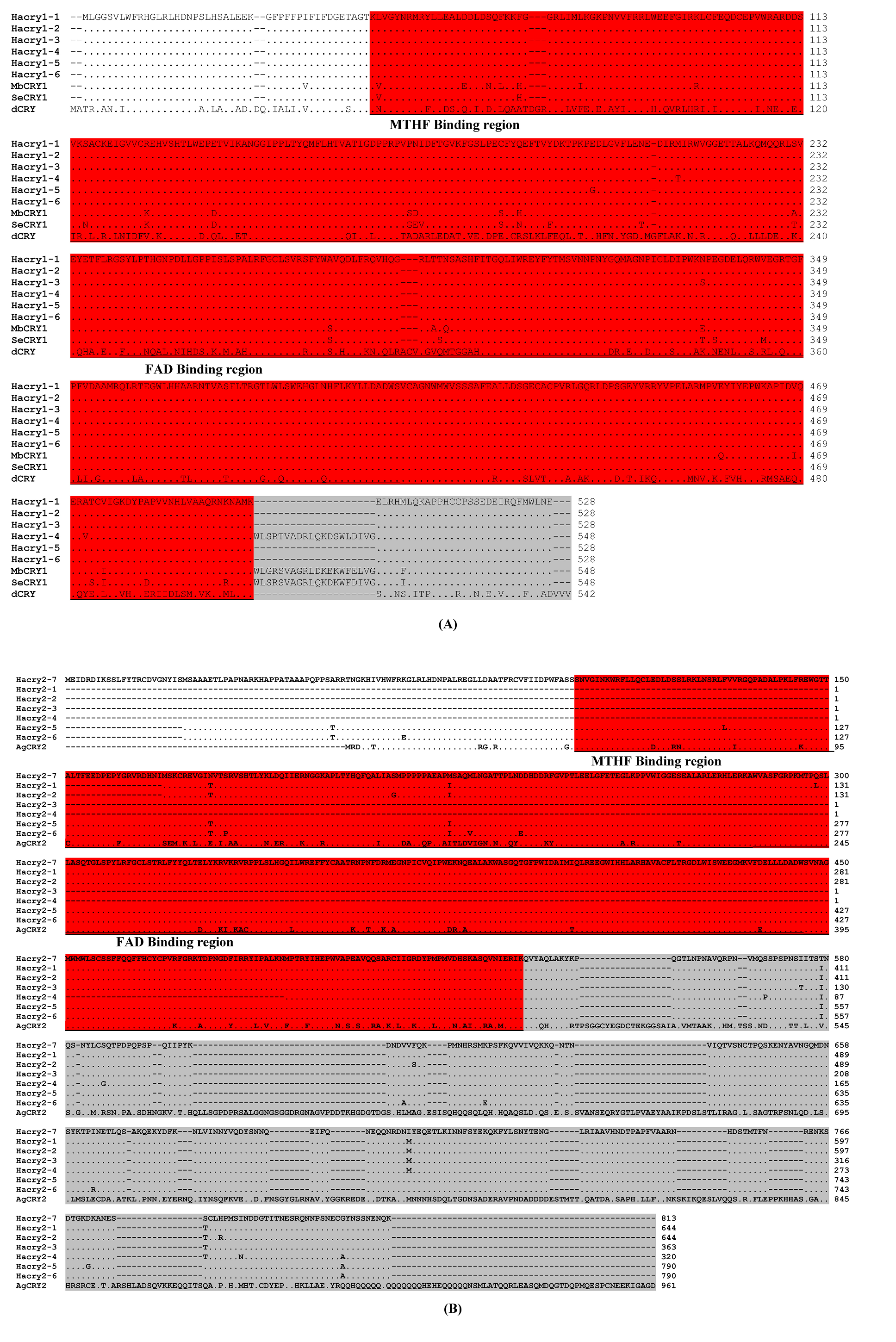

Supplement: Supplemental Information 5 — Region shaded in red correspond to the PHR region. Gray shown the CCE region. “—” stands for MTHF and FAD binding region, in which “=” stands for the shared region by MTHF and FAD. “.” stands for identical sites with the first row sequence. “-” stand for gap. dCRY: Drosphila melanogaster (accession no.: AAK92938), MbCRY1: Mamestra brassicae (accession no.: AAY23345), SeCRY1: Spodoptera exigua (accession no.: ADY17887), AgCRY2: Anopheles gambiae (accession no.: XP_313179). [file peerj-07-8071-s005.png]
